# Performance status eligibility requirements and enrollment characteristics in cancer clinical trials leading to US Food and Drug Administration drugs approval (2009–2023)

**DOI:** 10.1016/j.ejca.2025.115589

**Published:** 2025-06-26

**Authors:** Giovanni Maria Iannantuono, Charalampos S. Floudas, Marco Filetti, Tommaso Giovagnoli, Stefano Sganga, Antonio Vitale, Elias Chandran, Pasquale Lombardi, Roberto Rosenfeld, Elena Giudice, Elisabetta Xue, Elvira Rapisarda, Paola Troisi, Andrea B. Apolo, Fatima Karzai, Emilio Bria, James L. Gulley, Gennaro Daniele

**Affiliations:** a Phase 1 Unit, Fondazione Policlinico Universitario Agostino Gemelli IRCCS, Rome, Italy; b Center for Immuno-Oncology, Center for Cancer Research, National Cancer Institute, National Institutes of Health, Bethesda, MD, USA; c Comprehensive Cancer Center, Medical Oncology Department, Fondazione Policlinico Universitario Agostino Gemelli IRCCS, Rome, Italy; d Faculty of Medicine and Surgery, Università Cattolica del Sacro Cuore, Rome, Italy; e Genitourinary Malignancies Branch, Center for Cancer Research, National Cancer Institute, National Institutes of Health, Bethesda, MD, USA; f Department of Surgery and Cancer, Hammersmith Hospital Campus, Imperial College London, London, UK; g Precision Medicine in Senology Unit, Fondazione Policlinico Universitario Agostino Gemelli IRCCS, Rome, Italy; h Department of Woman, Child and Public Health, Fondazione Policlinico Universitario Agostino Gemelli IRCCS, Rome, Italy; i Institute of Obstetrics and Gynecology, Università Cattolica del Sacro Cuore, Rome, Italy; j Medical Oncology Unit, Ospedale Isola Tiberina-Gemelli Isola, Rome, Italy

**Keywords:** Performance status, Eligibility criteria, Clinical trial, Solid tumor

## Abstract

**Background:**

Participants with a low functional status are often excluded from cancer clinical trials, limiting the generalizability of their results. Here we aimed to investigate performance status (PS) eligibility requirements and enrollment characteristics in clinical trials leading to anticancer drug approvals.

**Methods:**

We conducted a cross-sectional study on pivotal clinical trials for non-hematologic solid tumors leading to drug approvals by the US Food and Drug Administration from 2009 to 2023. Participants with an Eastern Cooperative Oncology Group (ECOG) PS ≥ 2 were defined as having low functional status (i.e., poor PS).

**Results:**

We identified 283 clinical trials with 158,510 total participants. Four (1.4 %) studies did not use PS as an eligibility criterion. Of the remaining 279 trials, 72 (25.8 %) allowed the enrollment of poor-PS participants, with a negative trend over the 15-year interval (p = 0.01). The proportion of studies enrolling ECOG PS ≥ 2 participants was 43.2 % from 2009–2013, 29.6 % from 2014–2018, and 17.5 % from 2019–2023 (p = 0.002) Notably, early-phase studies included poor-PS participants more frequently than phase 3 clinical trials (40.8 % vs 20.2 %; p = 0.01). Finally, over the 15-year interval, the median (interquartile range) proportions of ECOG PS 0, 1, and 2 participants were 53.7 % (38.7 %–65.7 %), 45.1 % (33.5 %–58.8 %), and 4.3 % (1.8 %–7.9 %), respectively.

**Conclusions:**

A limited fraction of pivotal clinical trials included participants with poor PS, with a median percentage enrollment of less than 5 %. Sponsors, institutional review boards, and investigators must collaborate to broaden PS eligibility criteria to achieve more representative trial populations.

## 1. Introduction

Clinical trials, the cornerstone of evidence-based medicine, generate the information required to guide clinical and therapeutic decisions. Defining eligibility criteria is essential to shaping a clinical trial’s sample population and affects whether study results can be generalized to the population the study drug is targeting [1]. Performance status (PS) is one of the universal eligibility criteria used in clinical trials [2]. It describes participants’ functional level based on their ability to care for themselves, carry out daily activities, or perform physical tasks [3]. PS scales were first introduced in 1948 to evaluate the treatment effects of chemotherapy in cancer patients [4]. However, around the 1970s, the use of PS scales changed from assessing the efficacy of anticancer treatments to defining participants’ functionality for clinical trials, with the goal of reducing the heterogeneity of the enrolled population [5].

PS is a well-recognized prognostic factor in patients affected by advanced solid tumors [6,7]. Currently, various PS scales are available to identify patients with low- and high-functional states, including the Eastern Cooperative Oncology Group (ECOG) PS scale and the Karnofsky Performance Scale (KPS). Low KPS and high ECOG PS scores identify those patients with a worse or poor PS, respectively [4,8]. Participants with poor PS are usually excluded from clinical trials [9] because those with a low functional status have a higher risk of developing adverse events than participants with a better PS [10]. Furthermore, enrollment of participants with poor PS may have a detrimental impact on clinical trial outcomes [11]. Nevertheless, excluding poor-PS participants from clinical trials limits the generalizability of information obtained from these studies to the broader population that may ultimately receive the drug being investigated in clinical practice [9].

Jin et al. have shown that 60 % of the clinical trials submitted in 2015 as investigational new drug (IND) applications to the US Food and Drug Administration (FDA) allowed only participants with good PS (ECOG PS ≤1) to be enrolled [2]. In 2020, Abi Jaoude et al. demonstrated that in approximately 40 % of 600 phase 3 clinical trials of antitumor drugs eligibility was restricted to participants with an ECOG PS of ≤ 1. In the 156 clinical trials that led to subsequent FDA approval of the IND, this percentage increased to 45 % [12]. In 2021, the American Society of Clinical Oncology (ASCO)-Friends of Cancer Research Performance Status Work Group recommended that participants with an ECOG PS of ≥ 2 should be included in clinical trials unless there is a valid scientific or medical reason for exclusion supported by recognized safety concerns [10].

In recent years, there has been a trend toward regulatory agencies approving antitumor drugs based on earlier-phase clinical trials. Between 2012 and 2021, almost 60 % of anticancer drugs were FDA-approved on the basis of early-phase clinical trial results [13]. Here, we comprehensively evaluate the PS eligibility requirements and characteristics of enrolled participants in pivotal clinical trials that led to FDA approvals of antitumor drugs within the 15-year timeframe of 2009–2023.

## 2. Methods

We conducted a cross-sectional study of an extensive number of clinical trials that led to FDA approval of antitumor drugs to analyze the PS eligibility requirements and the characteristics of the enrolled participants. This study did not involve information on individual patients but exclusively relied on data from previously published clinical trials and, thus, did not require approval from an institutional review board. Furthermore, the study adhered to the Strengthening the Reporting of Observational Studies in Epidemiology (STROBE) reporting guidelines [14].

Initially, we reviewed approval notifications published during a 15-year interval from January 1, 2009 to December 31, 2023 in the “Oncology (Cancer)/Hematologic Malignancies Approval Notifications” section of the FDA website [15]. We included approvals related to new antitumor drugs for adults and children with solid tumors. Subsequently, we identified the pivotal clinical trials referenced in each selected approval. For each clinical trial, we retrieved the protocol, the manuscript reporting the data referenced in the approval notification (if available), and any supplementary materials. We extracted information on the study phase, the class of antitumor drug, the type of sponsor, the PS scale and scores used as eligibility criteria, and information related to the enrolled participants (absolute number, type of cancer, tumor staging, and previous treatments). Early-phase clinical trials were defined as phase 1, phase 1/2, or phase 2. Participants with good or poor PS were defined as ECOG PS ≤ 1 and ECOG PS ≥ 2, respectively. In the case of studies using KPS, the World Health Organization (WHO) scale, or the Gynecologic Oncology Group (GOG) scale, PS scores were converted to the ECOG PS scale based on available evidence in the literature [16].

### 2.1. Statistical analyses

Clinical trials’ PS eligibility requirements and enrollment characteristics were evaluated throughout the 15-year timeframe comprising 3 consecutive 5-year intervals (2009–2013, 2014–2018, 2019–2023). These assessments were performed for both the whole collection of clinical trials and the subgroups of early- and late-phase clinical trials. In addition, we compared the enrollment characteristics of clinical trials that led to approvals published before and after the release of the ASCO-Friends of Cancer Research Performance Status Work Group recommendations (2009–2020 vs 2021–2023).

Variables of interest were summarized through descriptive statistics, and results were reported as both absolute measures and proportions alongside interquartile ranges and standard deviations, as appropriate. Differences in proportions were assessed using Pearson’s chi-squared test or Fisher’s exact test. Differences in medians were evaluated by the Kruskal-Wallis test. Temporal trends were analyzed by performing Cuzick’s test for trend. Although there were no a priori hypotheses, all the analyses were considered statistically significant when p < 0.05. To account for multiple comparisons, Bonferroni corrections were applied to the analyses related to the whole collection of clinical trials. On the contrary, the analyses regarding the subgroups of early- and late phase clinical trials were not corrected for multiplicity due to their exploratory nature. The significance level after multiplicity correction was set at 0.05. All analyses and graphical representations were conducted using R Studio (version 4.4.0).

## 3. Results

Of the 535 approval notifications published from 2009–2023, we deemed 271 to be eligible for this study (Figure S1). From the selected approvals, 283 clinical trials were identified and 158,510 participants. Seventy-eight clinical trials (27.6 %) were early-phase; 205 (72.4 %) were phase 3. The other characteristics of the included clinical trials and their participants are reported in the Table S1.

### 3.1. PS eligibility requirements in the included clinical trials

Four clinical trials (1.4 %) published between 2012 and 2020 did not include PS among eligibility criteria. These four studies assessed antitumor drugs in relapsed or refractory high-risk neuroblastoma (NCT01757626) [17], tenosynovial giant cell tumor (NCT02371369) [18], unresectable/metastatic paraganglioma or pheochromocytoma (NCT00874614) [19], and subependymal giant cell astrocytoma in patients affected by tuberous sclerosis complex (NCT00789828) [20]. Of the remaining 279 clinical trials, 244 (86.2 %) used the ECOG PS scale, 17 (6.1 %) the KPS, 13 (4.7 %) the WHO scale, 2 (0.7 %) the GOG scale, and 3 (1.1 %) a combination of the KPS and Lansky scales.

**Fig. 1.**
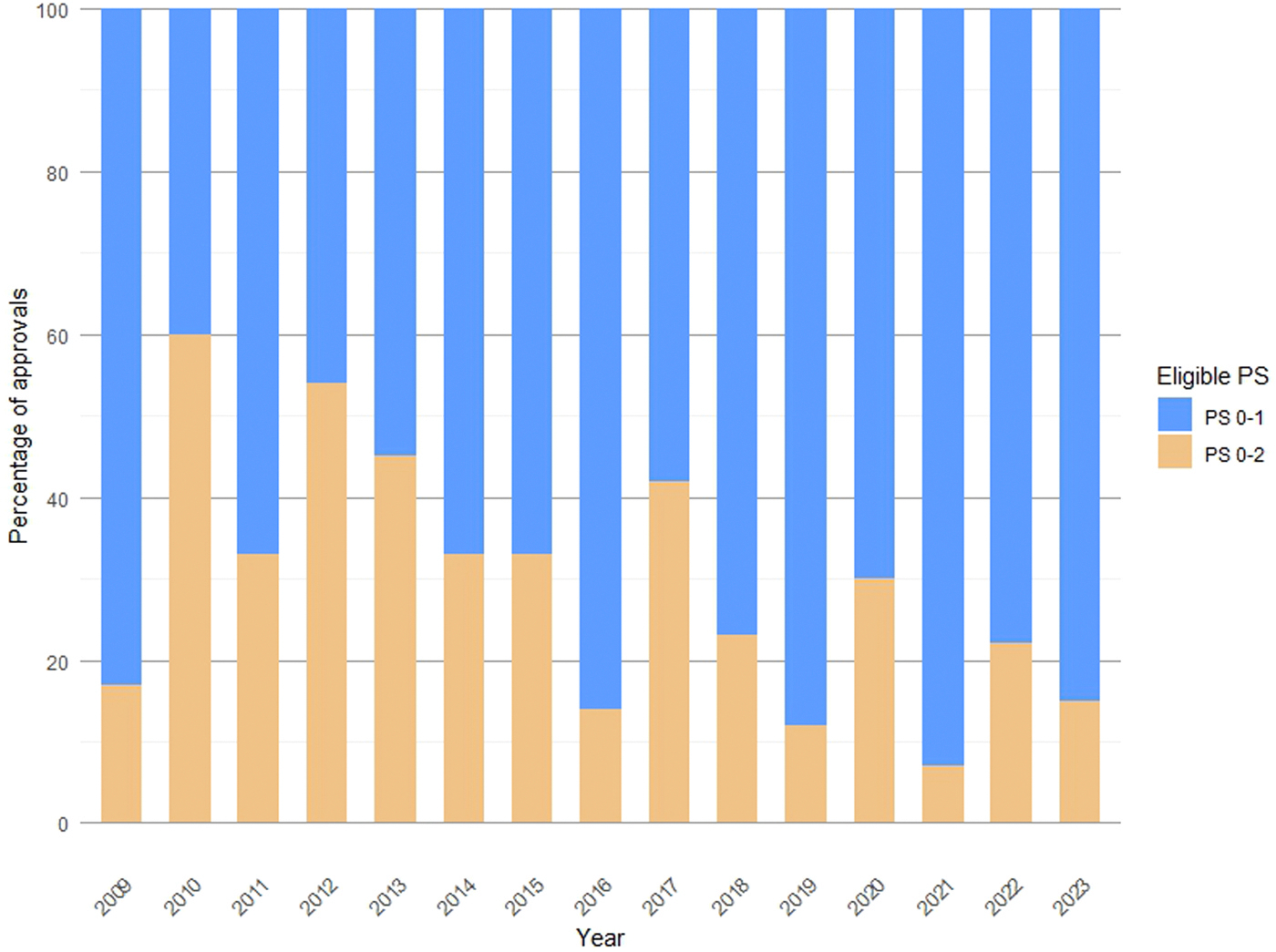
Proportion of clinical trials enrolling poor-PS participants over the 15-year time interval.

Within this group, 72 clinical trials (25.8 %) allowed the enrollment of poor-PS participants, with a negative trend over the 15-year timeframe (z = _–_2.31; p = 0.01) (Figure 1). Notably, the percent of studies enrolling ECOG PS ≥ 2 in the intervals 2009–2013, 2014–2018, and 2019–2023 were 43.2 %, 29.6 %, and 17.5 %, respectively (p = 0.002) (Table S2). In the subset of early-phase clinical trials, the highest percentage of studies permitting the inclusion of poor-PS participants was reported in 2014–2018 (45.2 %) compared to 2009–2013 (40 %) and 2019–2023 (37.5 %) (p = 0.87). In contrast, in the subset of phase 3 clinical trials, the proportion of studies enrolling ECOG PS ≥2 decreased over time (43.6 % vs 22.4 % vs 9.3 %; p < 0.001) (Table S2).

Early-phase studies included participants with poor PS more frequently than phase 3 clinical trials (40.8 % vs 20.2 %; p = 0.01). No statistically significant differences were found in terms of type of regimen, type of sponsor, tumor staging, and prior treatments (Table 1). Among the different tumor types, the enrollment of participants with poor PS was permitted more frequently in studies of endocrine tumors, soft tissue sarcomas, and neuroendocrine tumors, despite a low absolute number of clinical trials. Among the different classes of drugs, no clinical trials of antibody-drug conjugates and only 7.0 % of studies of immunotherapeutic agents allowed the enrollment of patients with ECOG PS ≥ 2 (Table 1).

### 3.2. Characteristics of enrolled participants in the included clinical trials

**Fig. 2.**
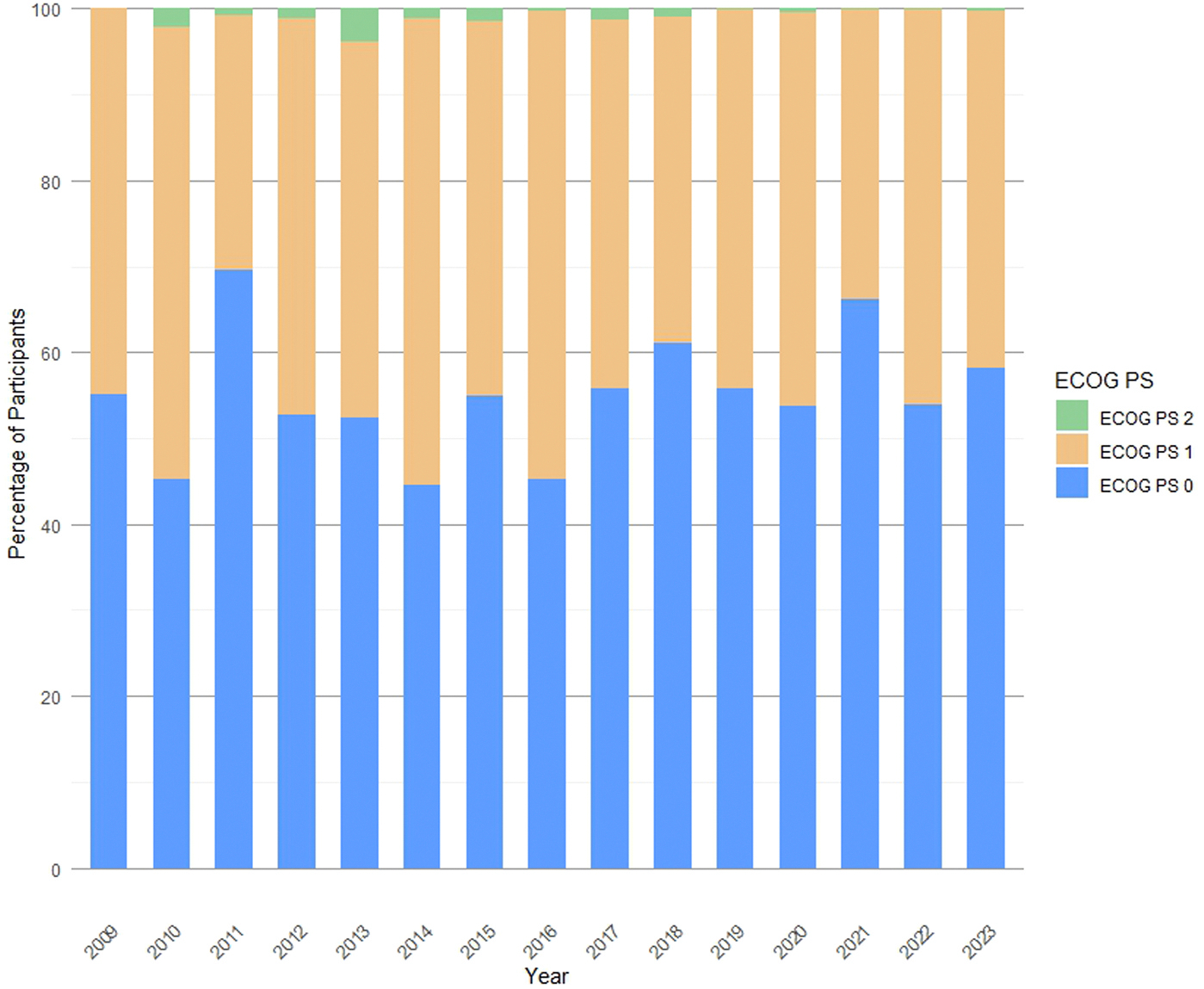
Proportion of enrolled poor-PS participants over the 15-year time interval.

Thirty-three clinical trials (11.8 %) were excluded from our analysis since 20 did not report the PS of enrolled participants and 13 did not specify the breakdown of participants according to PS (Table S3). Within this group, 89.4 % of clinical trials were phase 3, 69.7 % enrolled patients with metastatic tumors, and, most interestingly, more than half (57.6 %) allowed the inclusion of poor-PS participants. In the remaining 246 clinical trials (131,134 participants) over the 15-year timeframe, the median (interquartile range) proportions of ECOG PS 0, ECOG PS 1, and ECOG PS 2 participants were 53.7 % (range: 38.7 %–65.7 %), 45.1 % (range: 33.5 %–58.8 %), and 4.3 % (range: 1.8 %–7.9 %), respectively (Figure 2). The proportion of participants with each PS did not change significantly among the three 5-year intervals or among the subsets of early-phase and phase 3 clinical trials (Table S4).

Percent enrollment of patients with poor PS was similar among the different groups (range: 0.6 %–8.0 %). Notably, we found that 12.8 % of enrolled participants in clinical trials assessing radiopharmaceutical drugs had an ECOG PS ≥ 2 (Table 1). Finally, when considering a time landmark corresponding to the publication of the ASCO-Friends of Cancer Research recommendations [9], we found a lower percentage of enrolled poor-PS participants in the clinical trials leading to FDA approval after 2021 compared to those before 2021 (6.4 % vs 2.2 %; p = 0.01). A not statistically significant change was detected in the subsets of early-phase and phase 3 studies (Table S5).

## 4. Discussion

This study was the first comprehensive investigation of the PS eligibility requirements and enrollment characteristics of an extensive collection of pivotal cancer clinical trials. We reviewed 271 FDA approvals published from 2009–2023 and identified 283 clinical trials with 158,510 total participants. Our results confirm that enrollment of poor-PS participants was allowed in a limited proportion of clinical trials (25.8 %), with a negative trend over the 15-year timeframe covered by this study. In addition, we found a lower proportion of studies enrolling poor-PS participants in comparison to the data provided by Jin et al. on the IND applications submitted to the FDA in 2015 (25.8 % vs 35 %) [2]. Moreover, we found a higher percentage of phase 3 studies excluding participants with poor PS compared to data reported by Abi Jaoude et al. (79.8 % vs 45.5 %) [12]. Our results further confirm that participants with poor PS represent the lowest proportion of clinical trial participants (median 4.3 %).

The results of this study provide additional information on the use of restrictive eligibility criteria by offering an assessment of the PS eligibility spectrum within the context of different tumor types and systemic anticancer therapies. Regarding cancer types, it is noteworthy that only 24.6 % of pivotal clinical trials of lung cancer enrolled participants with poor PS (median 7.8 %), although the available evidence suggests that such patients represent 18 %–34 % of the general population [6,21]. Similarly, only 31 % of studies of colon cancer enrolled patients with low functional status (median 3.3 %), despite such participants representing 29 % of the population affected by colon cancer [22]. Regarding systemic anticancer therapies, it is also notable that only 7.0 % of clinical trials leading to the approval of immunotherapy-based agents enrolled participants with poor PS (median 0.6 %). Indeed, although the safety profile of immunotherapy agents is more favorable than that of chemotherapy [23], the proportion of studies enrolling ECOG PS ≥ 2 participants in trials of chemotherapy as a single-agent or in combination therapy was higher than in trials of immunotherapy agents (38.5 % and 19.4 %, respectively). In addition, our results showed that industry-sponsored studies permitted the inclusion of poor-PS participants less frequently than clinical trials funded by academic institutions, research hospitals, cooperative groups, or the government (24.2 % vs 53.3 %), although this result is not statistically significant, and the absolute number of non-industry-sponsored clinical trials included in this study is low (6 %).

This study suggests that excluding participants with low functional status from clinical trials is partly due to the restrictive requirements included by those who are involved in the design of the study protocol (sponsors, institutional review boards [IRBs], and principal investigators) and partly to clinical investigators who are not inclined to enroll poor-PS participants. There may be various reasons for these findings [12]. First, treatment-related adverse events (especially severe adverse events) are more frequent in participants with low functional status, leading to an increased risk of toxicity and, thus, a potentially reduced treatment-effect benefit [10]. s, poor-PS participants (especially older adult patients) may have a higher rate of polypharmacy or potentially inappropriate medications, increasing the risk of interactions with the experimental drug [24]. Third, both patients and clinicians may be hesitant to enroll study participants with low functional status. Adhering to the procedures of a clinical trial may be more challenging for poor-PS participants, leading to higher dropout rates and increased loss to follow-up [11].

Nevertheless, excluding poor-PS participants limits the generalizability of clinical trial results. In this context, in 2021 the ASCO-Friends of Cancer Research Performance Status Work Group recommended broadening PS eligibility criteria to achieve more representative trial populations [9]. Through a simulated trial, they demonstrated that when the proportion of ECOG PS ≥ 2 participants was relatively low, the impact on the estimated hazard ratio and statistical power remained minimal, even in cases where these patients did not experience any therapeutic advantage [9]. Despite the release of these recommendations, we have shown that the number of enrolled poor-PS participants did not increase after 2021. One can argue that these trials were designed and conducted well before 2021, but we can also say that the guidelines were not the start of the discussion, but rather, its natural end. We concede that implementing these recommendations will take time, but also that the recommended results will not be achieved without the collaboration of regulatory agencies, sponsors, IRBs, and, most importantly, investigators.

Regulatory agencies have already addressed this point. In 2024 the FDA released a guidance for sponsors, IRBs, and clinical investigators suggesting that poor-PS participants should be included unless a scientific and/or clinical justification supports their exclusion based on established safety concerns [25]. Indeed, PS eligibility criteria should align with the patient population for whom the treatment is expected to be used in clinical practice. Early clinical safety and efficacy data should inform decisions regarding PS eligibility for the specific investigational therapy, and later-phase trials should generally reflect the intended patient population, including those with low functional status, except when prior trials have demonstrated safety risks [25]. Furthermore, if there are concerns about enrolling poor-PS participants in a given trial, alternative trial designs should be considered, such as pre-specified cohorts with ECOG PS ≥ 2 participants that may be excluded from the primary analysis. These cohorts should be small and exploratory, with an incremental enrollment strategy to enable an early stopping rule based on emerging safety data. This approach facilitates the inclusion of these patients while allowing for the collection of safety data. Finally, additional functional assessments should be explored to better characterize the baseline functional status of patients and track changes over time [25].

## 5. Conclusions

This is the first study to comprehensively evaluate PS eligibility requirements and enrollment characteristics of pivotal clinical trials that have led to FDA approval of anticancer drugs. The number of studies allowing the inclusion of participants with poor PS is limited (25.8 %), with a median percentage enrollment of less than 5 %. Although scientific societies and regulatory agencies have already highlighted the need to broaden PS eligibility criteria to achieve a more representative trial population, it is essential that sponsors, IRBs, and investigators work together to reduce the barriers to enrollment of poor-PS participants. On the one hand, it is crucial for sponsors, principal investigators, and IRBs to carefully select eligibility criteria when designing clinical trials (especially phase 3 studies) to avoid unnecessarily restrictive requirements. On the other hand, clinical investigators must be more inclined to enroll participants with a low-functional status. This combined approach will help make pivotal clinical trial evidence increasingly relevant and applicable to the general population who will receive the studied drug in clinical practice.

## Supplementary Material

Supplemental

Appendix A. Supporting information

Supplementary data associated with this article can be found in the online version at doi:10.1016/j.ejca.2025.115589.

## Figures and Tables

**Table 1:** Proportions of clinical trials including poor-PS patients and enrolled poor-PS participants based on the studies’ characteristics.

	Clinical trials enrolling poor-PS participants / No. (%)	Participants with poor PS enrolled in clinical trials / No. (%)

**Study phase**		
Early (phase 1, 1/2, 2)	31/76 (40.8)^c^	151/3082 (4.9)
Phase 3	41/203 (20.2)^c^	767/15077 (5.1)
**Type of regimen**		
Monotherapy	55/186 (29.6)	510/9711 (4.8)
Combined therapy	17/93 (18.3)	408/8448 (5.3)
**Tumor staging**		
Metastatic	67/251 (26.7)	911/16895 (5.4)
Non-metastatic	5/28 (17.9)	7/1264 (0.6)
**Prior treatments**		
Perioperative	5/28 (17.9)	7/1264 (0.6)
Untreated	20/83 (24.1)	449/7901 (5.7)
Pretreated	47/167 (24.1)	462/8994 (5.1)
**Sponsor**		
Industry	64/264 (24.2)	738/15481 (4.8)
Non-industry	8/15 (53.3)	180/2678 (6.7)
**Cancer type**		
Agnostic	8/13 (61.5)^c^	19/583 (3.3)
Brain cancer	3/4 (75.0)^c^	2/102 (2.0)
Breast cancer	3/32 (9.4)^c^	73/1417 (5.2)
Colorectal cancer	4/13 (30.8)^c^	110/3352 (3.3)
Endocrine tumors	5/6 (83.3)^c^	27/762 (3.5)
Gastrointestinal (non-colorectal) cancer	3/30 (10.0)^c^	12/405 (3.0)
Genitourinary (non-prostate) cancer	8/30 (26.7)^c^	57/2443 (2.3)
Gynecological cancer	4/19 (21.1)^c^	178/2787 (6.4)
Head and neck cancer	0/6 (0)^c^	NA
Lung cancer	14/57 (24.6)^c^	252/3217 (7.8)
Melanoma	0/25 (0)^c^	NA
Neuroendocrine tumors	3/5 (60.0)^c^	12/410 (2.9)
Prostate cancer	7/20 (35.0)^c^	135/12647 (1.1)
Skin cancer	2/6 (33.3)^c^	18/226 (8.0)
Soft tissue sarcoma	7/10 (70.0)^c^	23/1122 (2.1)
Other^a^	1/3 (33.3)^c^	0/28 (0)
**Class of drugs**		
Antibody-drug conjugate	0/12 (0)^c^	NA
Chemotherapy	5/13 (38.5)^c^	85/1308 (6.5)
Chemotherapy + other agents^b^	7/36 (19.4)^c^	360/6560 (5.5)
Hormonal therapy	4/14 (28.6)^c^	NA^d^
Hormonal therapy + targeted therapy	1/10 (10.0)^c^	12/666 (1.8)
Immunotherapy	5/71 (7.0)^c^	8/1271 (0.6)
Radiopharmaceutical	3/3 (100)^c^	118/919 (12.8)
Targeted therapy	44/115 (38.3)^c^	305/6394 (4.8)
Other	3/5 (60.0)^c^	30/1041 (2.9)

a Includes three clinical trials involving participants affected by Merkel cell carcinoma, Kaposi sarcoma, and pleural mesothelioma.

b Includes targeted therapy, immunotherapy, or other chemotherapeutic agents.

c Statistically significant differences (after Bonferroni correction) were found when comparing the number of clinical trials according to the following characteristics: study phase (p = 0.01), cancer type (p = 0.01), and class of drugs (p = 0.01).

d None of the four clinical trials reported the breakdown of participants according to PS.
