# Supplementary material for: Performance status eligibility requirements and enrollment characteristics in cancer clinical trials leading to US Food and Drug Administration drugs approval (2009–2023)
Source: Eur J Cancer. Author manuscript; Available in PMC 2025 Aug 18. (PMC12359999; doi:10.1016/j.ejca.2025.115589)
Supplement: Supplemental [file NIHMS2101104-supplement-Supplemental.pdf]

SUPPLEMENTARY MATERIALS

Figure S1. Flowchart of approval notifications included in the study.

Table S1. Participants' tumor types and class of drugs investigated in clinical trials included in the study.

Table S2. PS eligibility requirements in the three 5-year time intervals for the whole collection of clinical trials (A), early-phase clinical trials (B), and phase 3 clinical trials (C).

Table S3. Characteristics of clinical trials (n = 33) not reporting the PS of enrolled participants.

Table S4. Median percentage of participants by PS in the three 5-year intervals for the whole collection of clinical trials (A), early-phase clinical trials (B), and phase 3 clinical trials (C).

Table S5. Median percentage of participants by PS pre- and post-2021 for the whole collection of clinical trials (A), early-phase clinical trials (B), and phase 3 clinical trials (C).

Figure S1. Flowchart of approval notifications included in the study.

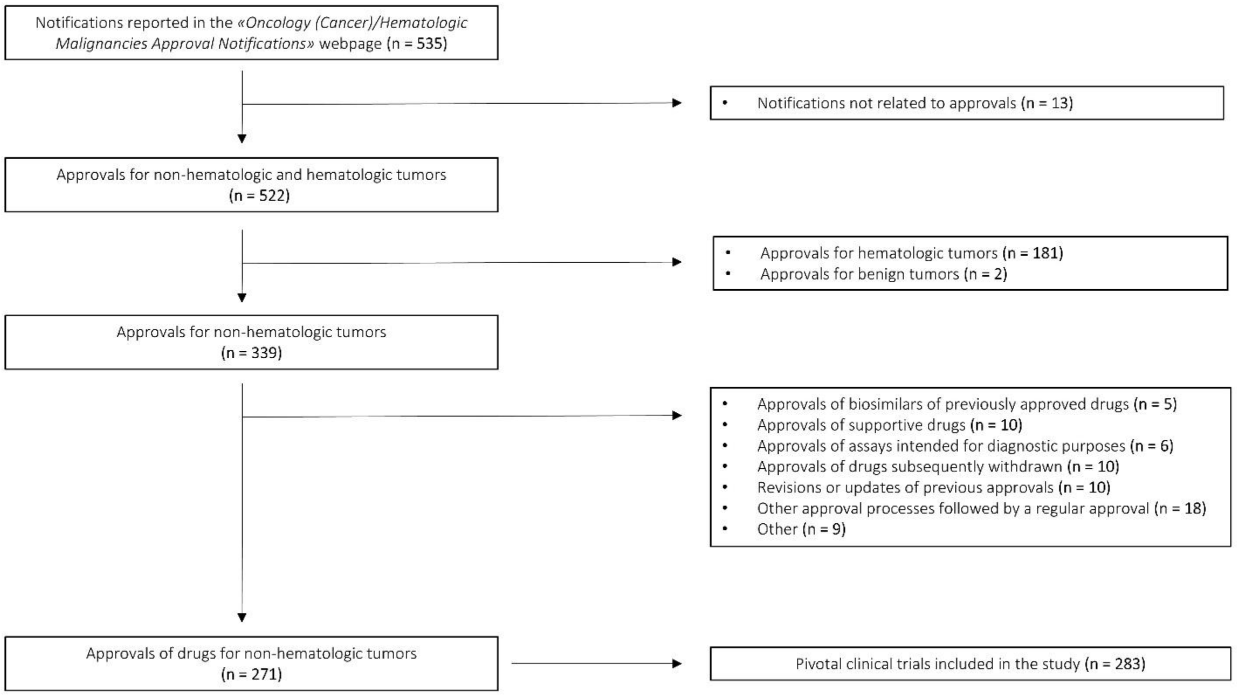

We excluded approvals that pertained to i) drugs for patients with solid and liquid hematologic malignancies; ii) drugs for patients with benign tumors; iii) biosimilars of previously approved drugs; iv) supportive drugs; v) assays intended for diagnostic purposes; vi) revision/update of indications regarding previously approved drugs; and vii) drugs subsequently withdrawn by the US Food and Drug Administration. In cases where a regular approval followed other approval processes (e.g., accelerated approval, breakthrough designation, etc.), we only included the regular approval.

Table S1. Participants' cancer types and class of drugs investigated in clinical trials included in the study.

**Cancer type**	**No. (%)**
Lung cancer	57 (20.1)
Breast cancer	32 (11.3)
Gastrointestinal (non-colorectal) cancer	30 (10.6)
Genitourinary (non-prostate) cancer	30 (10.6)
Melanoma	25 (8.8)
Prostate cancer	20 (7.1)
Gynecological cancer	19 (6.7)
Agnostic	13 (4.6)
Colorectal cancer	13 (4.6)
Soft tissue sarcoma	11 (3.9)
Head and neck cancer	6 (2.1)
Brain cancer	6 (2.1)
Skin cancer	6 (2.1)
Neuroendocrine tumors	6 (2.1)
Endocrine tumors	6 (2.1)
Other	3 (1.1)
**Class of drugs**	**No. (%)**
Targeted therapy	117 (41.3)
Immunotherapy	71 (25.1)
Chemotherapy + other agents	36 (12.7)
Hormonal therapy	14 (5.0)
Chemotherapy	13 (4.6)
Antibody-drug conjugates	12 (4.2)
Hormonal therapy + targeted therapy	10 (3.5)
Radiopharmaceutical	4 (1.4)
Other	6 (2.1)

Table S2. PS eligibility requirements in the three 5-year time intervals for the whole collection of clinical trials (A), early-phase clinical trials (B), and phase 3 clinical trials (C).

	Year	ECOG PS Eligibility No. (%)		P Value
		ECOG PS 0–1	ECOG PS ≥2	
A	2009–2013	25 (56.8)	19 (43.2)	0.002[a]
	2014–2018	69 (70.4)	29 (29.6)	
	2019–2023	113 (82.5)	24 (17.5)	
B	2009–2013	3 (60.0)	2 (40.0)	0.87[b]
	2014–2018	17 (54.8)	14 (45.2)	
	2019–2023	25 (62.5)	15 (37.5)	
C	2009–2013	22 (56.4)	17 (43.6)	<0.001[a]
	2014–2018	52 (77.6)	15 (22.4)	
	2019–2023	88 (90.7)	9 (9.3)	

Abbreviations: ECOG = Eastern Cooperative Oncology Group; PS = performance status.

[a]Based on chi-squared test.
[b]Based on fisher's exact test.

Table S3. Characteristics of clinical trials (n = 33) not reporting the PS of enrolled participants.

	**Clinical trials / No. (%)**
**Year**	
2009	1 (3.0)
2010	2 (6.1)
2011	2 (6.1)
2012	5 (15.2)
2014	1 (3.0)
2015	4 (12.1)
2017	4 (12.1)
2018	3 (9.1)
2019	5 (15.2)
2020	4 (12.1)
2022	2 (6.1)
**Study phase**	
Early (phase 1, 1/2, 2)	5 (15.2)
Phase 3	28 (84.9)
**Eligible PS**	
ECOG PS 0-1	14 (42.4)
ECOG PS ≥2	19 (57.6)
**Type of regimen**	
Monotherapy	24 (72.7)
Combined treatment	9 (27.3)
**Tumor staging**	
Metastatic	23 (69.7)
Non metastatic	10 (30.3)
**Prior treatments**	
Perioperative	10 (30.3)
Pretreated	15 (45.5)
Untreated	8 (24.2)
**Cancer types**	
Breast cancer	7 (21.2)
Endocrine tumors	2 (6.1)
Neuroendrocine tumors	2 (6.1)
Gastrointestinal (non-colorectal) cancer	2 (6.1)
Genitourinary (prostate) cancer	6 (18.2)
Lung cancer	2 (6.1)
Prostate cancer	3 (9.1)
Soft tissue sarcoma	2 (6.1)
Skin cancer	1 (3.0)
Brain cancer	2 (6.1)
Head and neck cancer	1 (3.0)
Melanoma	3 (9.1)

**Class of drugs**	
Chemotherapy	2 (6.1)
Chemotherapy with other agents	3 (9.1)
Hormonotherapy	5 (15.2)
Radiopharmaceutical	2 (6.1)
Targeted therapy	14 (42.4)
Immunotherapy	5 (15.2)
Antibody-drug conjugate	1 (3.0)
Other	1 (3.0)

Table S4. Median percentage of participants by PS in the three 5-year intervals for the whole collection of clinical trials (A), early-phase clinical trials (B), and phase 3 clinical trials (C).

	Participants by ECOG PS	Median % (IQR)	P value
A	**ECOG PS 0**		0.66[a]
	2009–2013	55.2 (42.0–64.5)	
	2014–2018	50.9 (35.9–66.7)	
	2019–2023	52.3 (39.0–65.5)	
	**ECOG PS 1**		0.68[a]
	2009–2013	43.1 (34.3–53.7)	
	2014–2018	44.7 (32.7–59.5)	
	2019–2023	46.3 (34.5–59.8)	
	**ECOG PS ≥2**		0.64[a]
	2009–2013	4.2 (3.0–8.3)	
	2014–2018	6.4 (1.1–8.5)	
	2019–2023	3.7 (1.9–6.7)	
B	**ECOG PS 0**		0.43[a]
	2009–2013	46.2 (43.8-67.2)	
	2014–2018	45.4 (33.2-54.8)	
	2019–2023	39.5 (33.1–56.6)	
	**ECOG PS 1**		0.51[a]
	2009–2013	53.8 (32.7-56.2)	
	2014–2018	54.0 (40.4-60.7)	
	2019–2023	56.2 (43.4–66.1)	
	**ECOG PS ≥2**		0.66[a]
	2009–2013	NA	
	2014–2018	7.8 (1.1–8.8)	
	2019–2023	5.2 (2.1–7.6)	
C	**ECOG PS 0**		0.62[a]
	2009–2013	55.3 (42.0-64.3)	
	2014–2018	57.1 (38.5-69.9)	
	2019–2023	58.1 (44.7–67.7)	
	**ECOG PS 1**		0.94[a]
	2009–2013	43.1 (34.5-52.5)	
	2014–2018	40.0 (29.9-57.6)	
	2019–2023	41.9 (31.3–54.4)	
	**ECOG PS ≥2**		0.36[a]
	2009–2013	4.2 (3.0–8.3)	
	2014–2018	3.5 (1.1–6.4)	
	2019–2023	2.9 (1.6–4.2)	

Abbreviations: ECOG = Eastern Cooperative Oncology Group; PS = performance status.

[a]Based on Kruskal-Wallis test.

Table S5. Median percentage of participants by PS pre- and post-2021 for the whole collection of clinical trials (A), early-phase clinical trials (B), and phase 3 clinical trials (C).

	Participants by ECOG PS	Median % (IQR)	P value
A	**ECOG PS 0**		0.47[a]
	2009–2020	52.9 (38.6–65.7)	
	2021–2023	55.9 (39.6–66.6)	
	**ECOG PS 1**		0.90[a]
	2009–2020	45.6 (33.9–58.7)	
	2021–2023	43.9 (33.3–59.9)	
	**ECOG PS ≥2**		0.01[a]
	2009–2020	6.4 (2.7–8.6)	
	2021–2023	2.2 (1.0–2.9)	
B	**ECOG PS 0**		0.52[a]
	2009–2020	42.0 (35.4-54.5)	
	2021–2023	37.9 (30.4–58.4)	
	**ECOG PS 1**		0.26[a]
	2009–2020	53.9 (43.0-59.7)	
	2021–2023	60.0 (41.6–69.0)	
	**ECOG PS ≥2**		0.05[a]
	2009–2020	7.7 (4.3–9.3)	
	2021–2023	1.9 (1.8–2.6)	
C	**ECOG PS 0**		0.20[a]
	2009–2020	55.9 (39.7-67.3)	
	2021–2023	58.9 (45.4–70.1)	
	**ECOG PS 1**		0.42[a]
	2009–2020	42.5 (31.7-56.1)	
	2021–2023	41.0 (29.7–54.3)	
	**ECOG PS ≥2**		0.09[a]
	2009–2020	4.3 (2.4–6.9)	
	2021–2023	2.6 (0.7–2.9)	

Abbreviations: ECOG = Eastern Cooperative Oncology Group; PS = performance status.

[a]Based on Kruskal-Wallis test.
